# Supplementary material for: Tree ring isotopes reveal an intensification of the hydrological cycle in the Amazon
Source: Commun Earth Environ. 2025 Jun 17;6(1):453. doi: 10.1038/s43247-025-02408-9 (PMC12173935; doi:10.1038/s43247-025-02408-9)
Supplement: Supplementary file 2 — Supplementary Materials [file 43247_2025_2408_MOESM2_ESM.pdf]

Supplementary Materials for

Tree ring isotopes reveal an intensification of the hydrological cycle in the Amazon

Bruno B. L. Cintra,* Emanuel Gloor, Jessica C. A. Baker, Arnoud Boom, Jochen Schöngart, Santiago Clerici, Kanhu Pattnayak, Roel J. W. Brienen

*Corresponding author. Email: b.ladvocat@bham.ac.uk

This file includes:

Supplementary Discussion
Supplementary Figures 1 to 7
Supplementary Tables 1 to 2
Supplementary References

Supplementary Discussion

Assessment and quantification of uncertainties to estimates of rainfall changes inferred from trends in $\delta^{18}O_{TR}$ from 1980 to 2010

Contribution of leaf evaporation to tree ring $\delta^{18}O$ signals

A large part of the signal of $\delta^{18}O_{TR}$ comes from variation in precipitation $\delta^{18}O$ caused by climate variation. However, $\delta^{18}O_{TR}$ is also affected by evaporative enrichment of transpiring leaves [1]. Higher leaf temperatures and vapor pressure deficit (VPD) increase leaf water enrichment, thus altering the original precipitation $\delta^{18}O$ signal [1–3]. This effect could be particularly strong for *M. acaciifolium* trees which grow during the driest months of the year. To address the possible effect of leaf water enrichment on the $\delta^{18}O_{TR}$ chronologies, we estimated the magnitude of the effects of both evaporative enrichment and stomatal conductance (g_s) responses to vapor pressure deficit using existing isotope models [1,4–7]. Those models are listed in Supplementary Table 2. Our analysis indicates that average contributions of leaf water enrichment to inter-annual variability in the $\delta^{18}O_{TR}$ record are 0.07±0.06‰ for the dry season chronology and 0.12±0.13‰ for the wet season chronology. These values represent approximately 10-12% of the interannual $\delta^{18}O_{TR}$ variation within each chronology. This analysis does not show evidence for these effects to cause consistent long-term trends (Supplementary Figure 9). These results agree with previous research for these species [8–10] showing that variation in $\delta^{18}O_{TR}$ reflects predominantly a signal of source water $\delta^{18}O$ with only weak influence of local temperature or VPD on $\delta^{18}O_{TR}$. In this analysis, we did not consider potential reductions in leaf stomatal conductance over time due to rising atmospheric CO_2 concentrations [11].

Evaporative enrichment of soil water

We assumed in our analysis that variation in tree ring $\delta^{18}O_{TR}$ largely reflected variation in rainwater $\delta^{18}O$ and not variation in soil evaporative enrichment. Soil water evaporative enrichment primarily occurs in the topsoil and is driven by the soil-to-air vapor pressure difference [12]. Due to high vapour pressure of tropical forest understories, evaporation from the

top soil is minimal and most of water vapor is derived from transpiration of plants [13]. As plant transpiration does not cause isotopic fractionation [14], evaporative enrichment of soil water is expected to be limited under the canopy of tropical forests. Furthermore, for soil water evaporative enrichment to exhibit temporal trends, substantial changes in the soil-to-air vapor pressure difference would be required. Given that atmospheric VPD has remained stable at our sites during the tree growing season (Supplementary Figure 8), it is unlikely that the soil-to-air vapor pressure gradient has changed significantly over time, and we thus conclude that it is unlikely that our $\delta^{18}O_{TR}$ records are influenced by changes in the degree of evaporative enrichment of soil water.

Changes in biochemical fractionation during synthesis of carbohydrates

The $\delta^{18}O$ of tree ring cellulose in our study is about 30‰ higher than the $\delta^{18}O$ of rainfall. This large difference results from isotopic enrichment of leaf water and biochemical fractionation (Epsilon bio) during the synthesis of carbohydrates and cellulose. On average, the difference between $\delta^{18}O_{TR}$ and $\delta^{18}O$ from source water ($\Delta^{18}O$) is approximately 27‰ [5,15].While the fractionating effect of biochemical reactions may be temperature dependent, this effect has shown to be very small in warm climates [16]. Over the analysed period in our study, Amazon temperature has increased by approximately 0.6°C, which would decrease $\Delta^{18}O$ (and consenquently cellulose $\delta^{18}O$) by 0.045‰ according to the temperature $\delta^{18}O$-relationship found by reference [16]. In comparison, the wet season decrease in $\delta^{18}O$ is 0.9‰, and any potential temperature effect of biochemical fractionation on our longer-term trends is thus very small.

Changes in $\delta^{18}O$ of moisture inflow

Secondly, we assumed that the isotopic composition of the vapour ($\delta^{18}O_{VAP}$) entering the Basin has remained unchanged over the analysed period. Increasing sea surface temperatures in the tropical Atlantic will decrease sea-to-air equilibrium fractionation during evaporation and increase $\delta^{18}O_{VAP}$. The 0.5°C increase in Atlantic Ocean SST's over the study period results in a small increase in incoming $\delta^{18}O_{VAP}$ of approximately 0.04‰ with only small effects on predictions in rainfall trends (Table 1, case 2).

Rainfall type and intensity

It has long been known that $\delta^{18}O$ in precipitation is affected by precipitation intensity [17,18] and precipitation type (e.g. stratiform vs convective) [19]. For the Amazon, some studies report an increase in the frequency of heavy precipitation events [20], which could affect rainfall $\delta^{18}O$, even when total rainfall stays the same. However, there is much spatial variability of trends for different regions within the basin [21], and we lack data to assess these effects. For this reason we assumed that there have been no changes in the rainfall type or intensity.

Additional Rayleigh distillation parameters

We also model the sensitivity of our precipitation change estimates to two other factors, the magnitude of the continental difference of $\delta^{18}O_{coast}$ to $\delta^{18}O_{site}$, and the assumed recycling ratio of evapotranspiration over precipitation, $r_{E:P}$ (see Methods). While our initial estimates of coast to site difference in $\delta^{18}O$ were based on $\delta^{18}O$ in precipitation measured at Global Network of Isotopes in Precipitation (GNIP) stations (see methods), we also used differences from the isotope enabled Hadley Centre Climate model [22], which results in slightly weaker changes in rainfall for both seasons (Table 1, case 7). Finally, our analysis shows that changing the assumed recycling ratio ($r_{E:P}$) by 10% resulted in increases or decreases in rainfall predictions by 1% (Table 1, case 8).

[13]

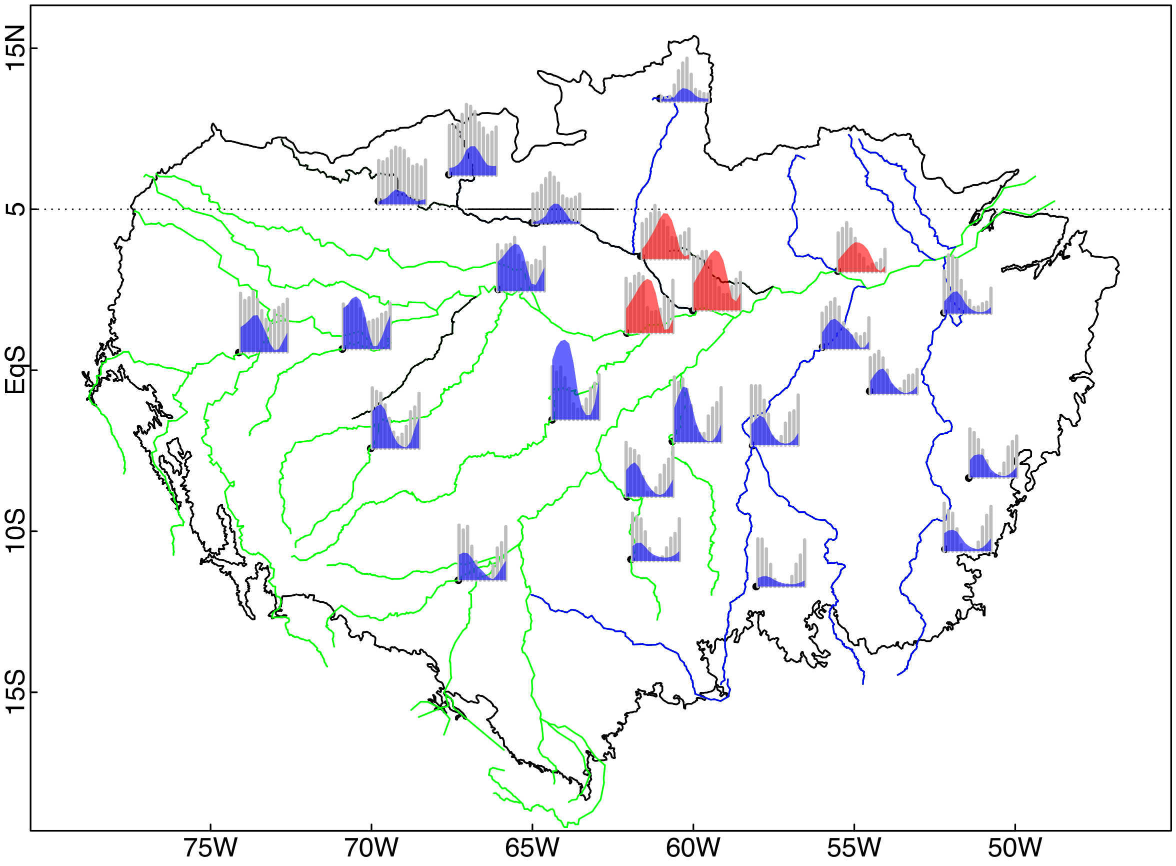

Supplementary Figure 1. Synchronicity between rainfall and river seasonality varies across the Amazon Basin. Map of the Amazon basin highlighting the rivers with blackwater (black lines), "white" water originating in the Andes (green lines) and clear water originating in the Brazilian and Guiana shields (blue lines). Pluviographs and hydrographs show the seasonality in precipitation (gray bars) and river level (blue shading) for various rivers across the basin. Red hydrographs indicate locations where the seasonality of the river levels lags the seasonality of local rainfall by 2 or more months. Data from hydroclimatic stations from the Brazilian Water Agency (ANA), from 1990 to 2015 A.D. Figure modified from [23]

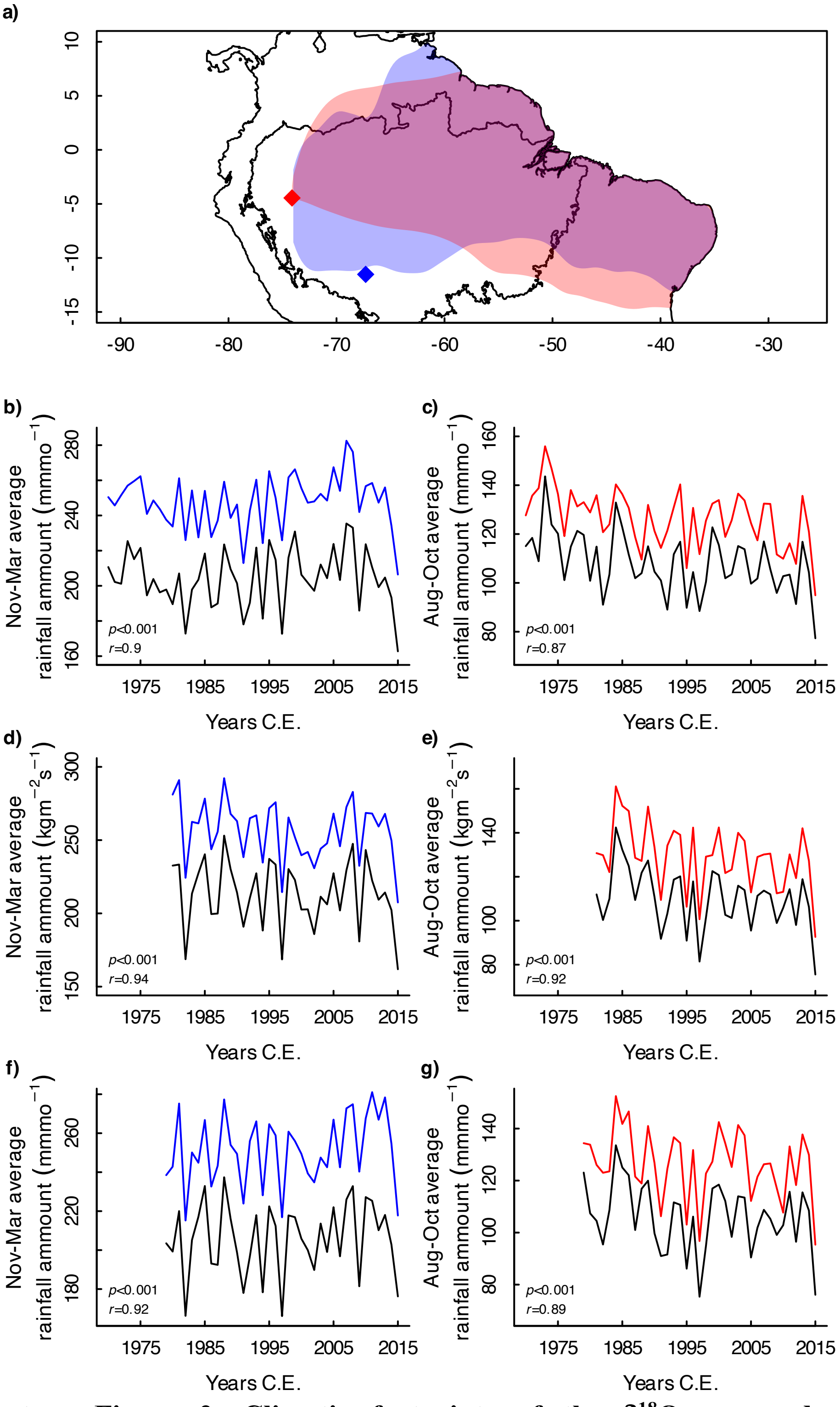

Supplementary Figure 2. **Climatic footprints of the $\delta^{18}O_{TR}$ records are highly representative of Amazon rainfall.** **(a)** Upwind regions of moisture transport to each sampling site, calculated from the HYSPLIT trajectories for the peak of the wet season (blue shading) and dry season (red shading). **(b,d,f)** Comparison of the wet season rainfall averaged over the blue region in **(a)** and over the Amazon region (inner black contour[24,25]), extracted from CRU **(b)**, CHIRPS **(d)** and MSWEP **(f)**. **(c,e,g)** As in **(b,d,f)**, but for the dry season and the red shaded area in **(a)**. In each panel from **(b-g)** the Pearson's correlation coefficient

between the two series is shown. Shaded regions in **(a)** were derived from the HYSPLIT back trajectories in Supplementary Figure 3 – see Methods section from main text for details.

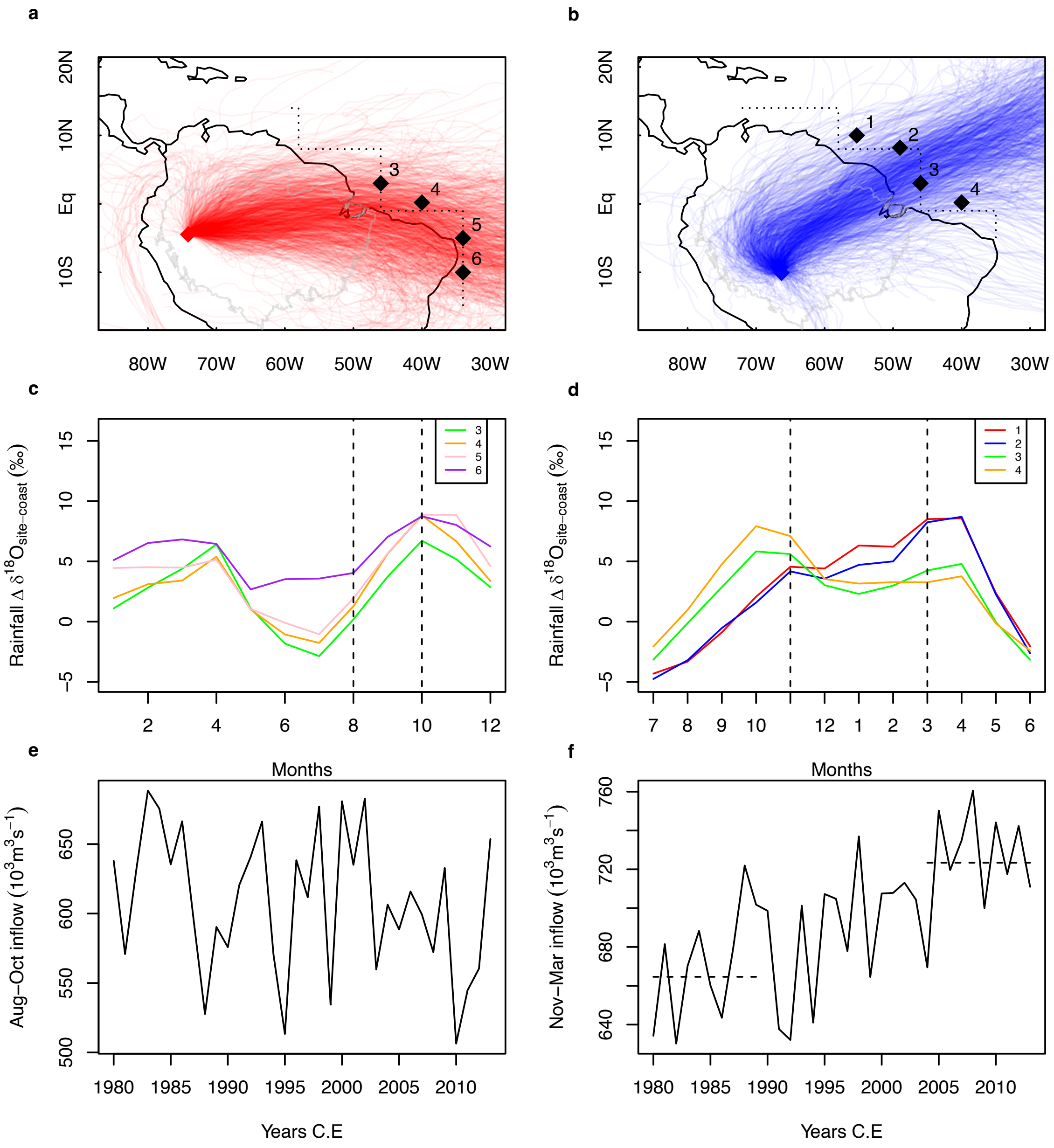

Supplementary Figure 3. Atmospheric and isotopic modelling and data were used to obtain rainfall $\delta^{18}O$ and moisture influx data for the Rayleigh distillation calculations from Equation [2]. Panels to the left and right show data for dry and wet season months, respectively. **(a,b)** Map of the Amazon basin[24,25]. HYSPLIT back trajectories from the sampling site (color diamonds) are shown as solid lines, with red and blue indicating sites and trajectories where trees grow during the dry and wet and seasons, respectively. The geographical range where trajectories cross the coast was used to position the boundary for moisture influx calculations (black dotted lines), and to define sea locations from where to obtain rainfall $\delta^{18}O$ (black diamonds). **(c,d)** $\Delta\delta^{18}O$ from the sites to the each sea location (diamonds in a,b). Line number in **(c,d)** indicates from which sea locations in **(a,b)** the $\Delta\delta^{18}O$ was calculated from. **(e,f)** Time series of moisture influx to the Amazon Basin estimated with equation [2] using ERA5 vertical integral of vapor flux, across the boundary shown as black

dotted lines in **(a,b)**. In **(c,d)**, vertical dashed lines indicate the period which was averaged to calculate the mean $\Delta\delta^{18}O$ and moisture influx for each site.

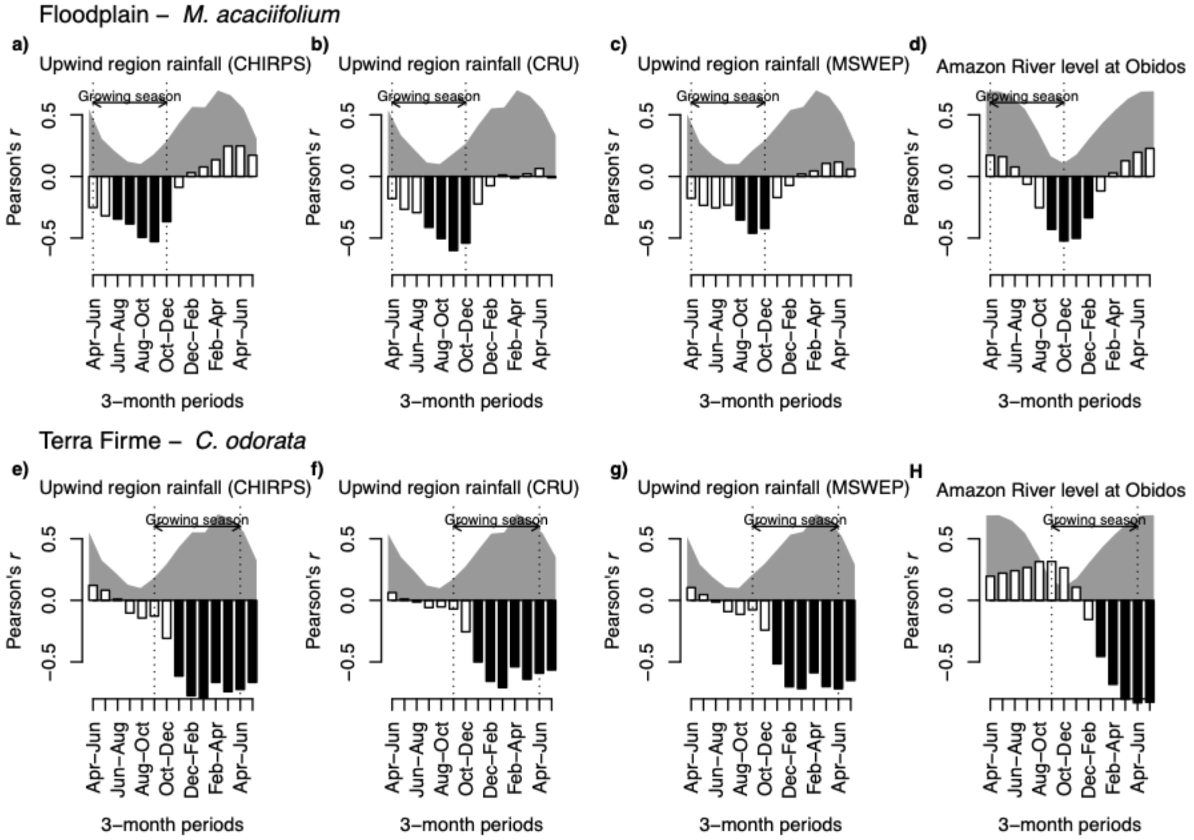

Supplementary Figure 4. $\delta^{18}O_{TR}$ records are correlated with inter-annual variation of hydroclimatic variables in the upwind regions of moisture transport. (a-d) Correlations with the $\delta^{18}O_{TR}$ records of the floodplain trees. **(e-h)** Correlations with the $\delta^{18}O_{TR}$ records of the terra firme trees. Bars indicate the Pearson's *r* between $\delta^{18}O_{TR}$ and 3-monthly means of rainfall or river levels. Filled bars indicate significant correlations with 95% confidence interval. Shaded areas show seasonal variation in precipitation for the upwind regions **(a,b,c,e,f,g)** or Amazon river levels at Obidos **(d,h)**. Dotted lines and arrows indicate the growing season of the floodplain and terra firme trees.

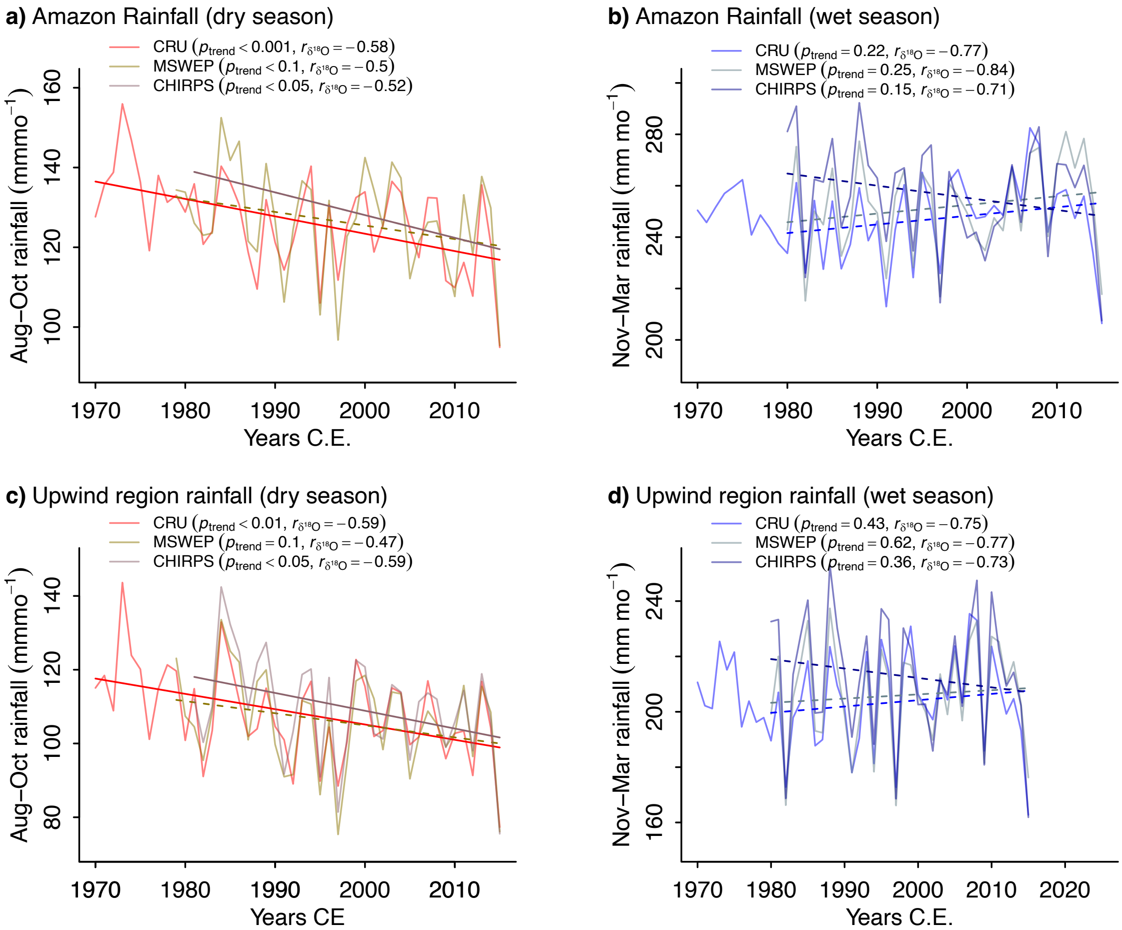

Supplementary Figure 5. Trends in rainfall amounts (p_{trend}) and Pearson's correlations with $\delta^{18}O_{TR}$ ($r_{\delta18O}$) are very similar for rainfall averaged over the entire Amazon basin and over the upwind regions of moisture transport. Time series of rainfall were obtained obtained from CRU, MCWEP (scale on left axis) and CHIRPS (scale on right axis) for dry **(a,c)** and wet **(b,d)** seasons. All correlations with $\delta^{18}O_{TR}$ were done with the terra firme site trees for the wet season and with the floodplain site trees for the dry season (see Figure 1 in the main text). Upwind rainfall regions correspond the shaded regions in Figure 1a and Supplementary Figure 2 – see Methods section from main text for details.

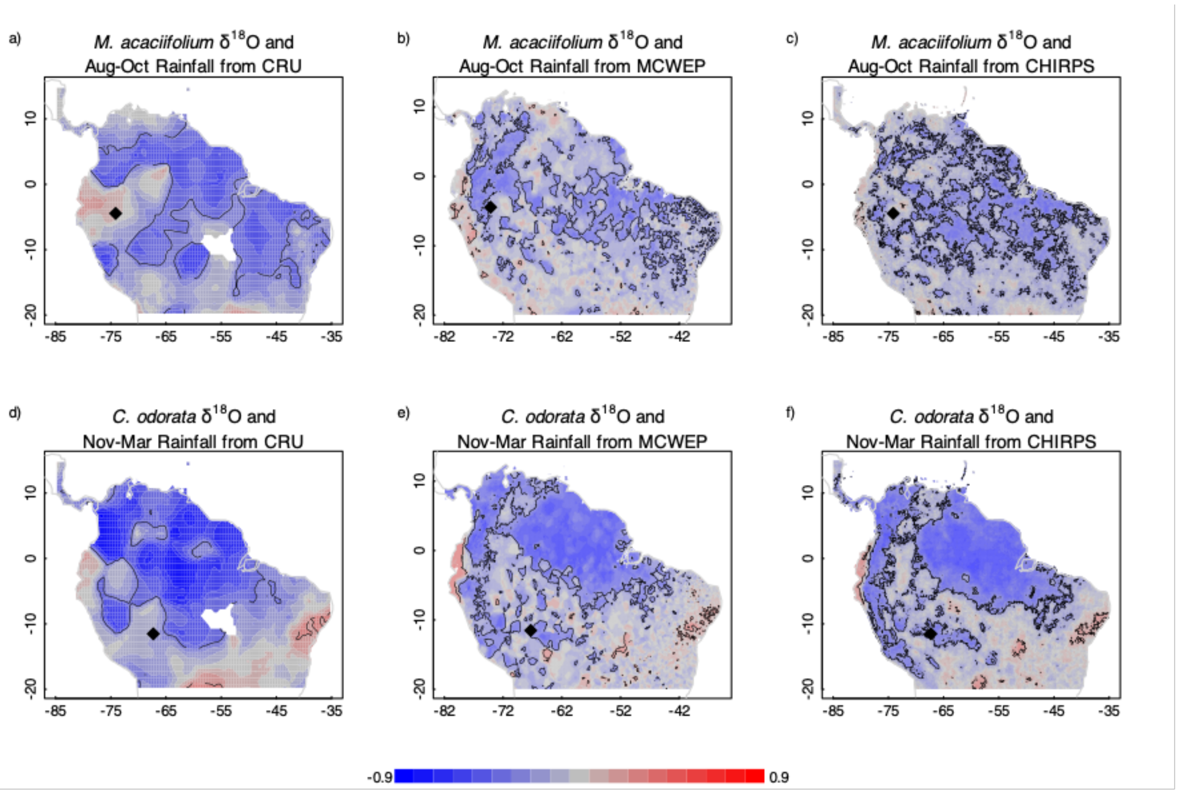

Supplementary Figure 6. Spatial correlation maps of the $\delta^{18}O_{TR}$ records with gridded precipitation datasets confirm the large-scale climatic footprint of the $\delta^{18}O_{TR}$ records. Color scale indicates the Pearson's correlation coefficient. Contours show regions of significant correlations ($p<0.1$). All correlations were computed for 1980 onwards.

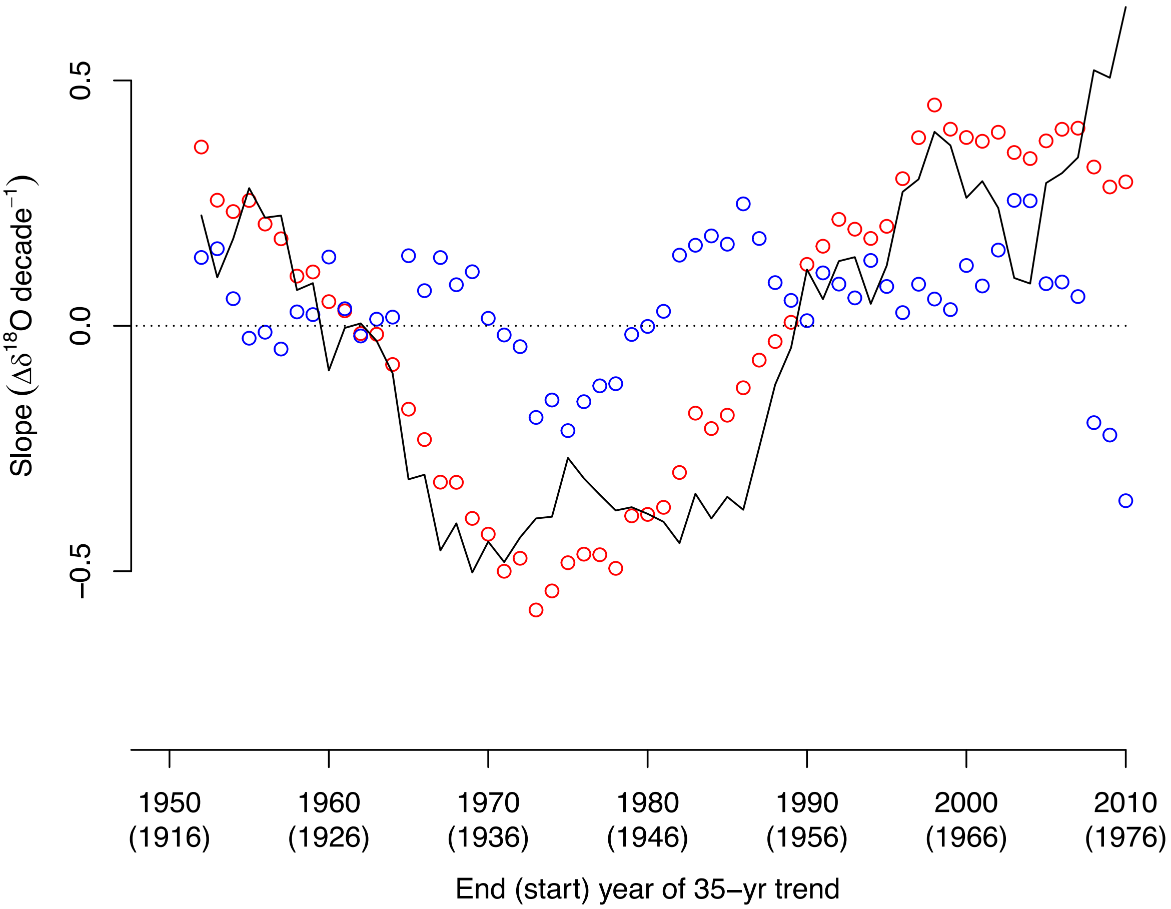

Supplementary Figure 7. The divergence between the two records since mid 1970s is greater than at any other time in the period covered. $\delta^{18}O$ trend slopes for sliding windows of 35ys in the C. odorata (blue, wet season) and M. acaciifolium (red, dry season) $\delta^{18}O$ records. Soid line indicates the difference between the trend slopes of the two records.

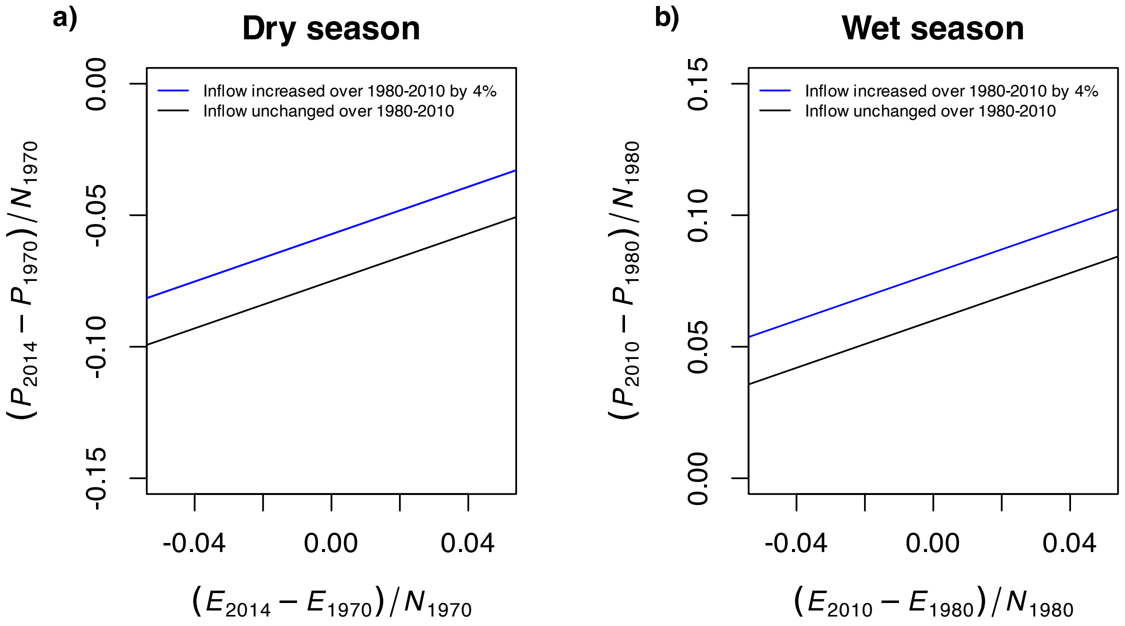

Supplementary Figure 8. **Estimated changes in rainfall amounts inferred from the Rayleigh distillation model can be expressed as fractions of the total moisture inflow at the start of the analyzed period.** Same as in main text Figure 3 **(d,e)** but with changes in rainfall and evapotranspiration expressed as a fraction of total moisture influx in 1980.

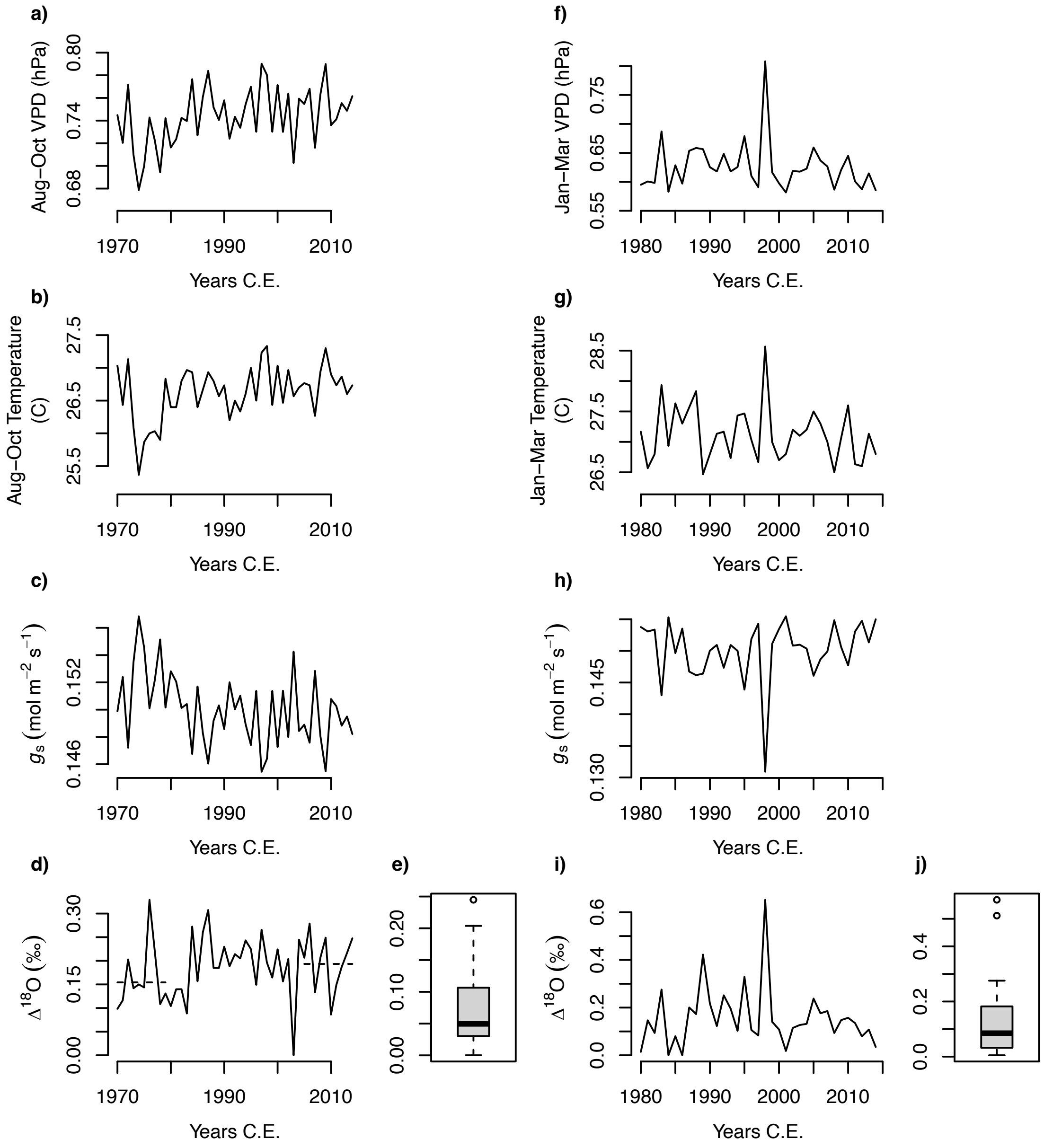

Supplementary Figure 9. **Long-term variation in local climate conditions during the months of strongest correlation with $\delta^{18}O_{TR}$ result in small changes of modelled leaf water enrichment levels.** Panels on the left show variables for the dry season record of the floodplain site. Panels on the right show variables for the wet season record of the *terra firme* site. **(a,f)** Vapor pressure deficit, **(b,g)** temperature, **(c,h)** stomatal conductance (g_s), **(d,i)** the contribution of leaf water enrichment to inter-annual variation in tree ring $\delta^{18}O_{TR}$ ($\Delta^{18}O$). **(e,j)** Box plots indicate the inter-annual variability from **(d,i)** as the difference between the $\Delta^{18}O$ of consecutive years, e.g. $\Delta^{18}O_t$ - $\Delta^{18}O_{t-1}$. g_s values from **(c,h)** were calculated as a function of VPD, $g_{s.t}$= g_{s0}*(1/1+(VPD_t/VPD_{mean})), with $g_{s.0}$=0.3 mol m^{-2} s^{-1} the assumed maximum g_s and VPD_{mean} the long-term VPD mean, e.g. the average values of the time series from **(a,f)**.

Supplementary Table 1. Parameters used for Case 0 of estimates of rainfall changes (Table 1 from main text) were based on rainfall $\delta^{18}O$ and variables influencing its fractionation during moisture transport.

	Dry season		Wet season	
Parameters	Value	Source	Value	Source
Months of the year	Aug-Oct	See main text Figure 1	Nov-Mar	See main text Figure 1
Station $\delta^{18}O_{site}$	-4.41‰	GNIP - Puerto Almendras - Iquitos	-6.1‰	Riberalta, Bolivia (this study)
Model $\delta^{18}O_{site}$	-6.41‰	HadAM3 [22]*	-9.28‰	HadAM3 [22]*
Station $\delta^{18}O_{coast}$	-0.87‰	GNIP - Belém	-2.282‰	GNIP - Belém
Model $\delta^{18}O_{coast}$	-1.19‰	HadAM3 [22]*	-4.58‰	HadAM3 [22]*
$\delta^{18}O_{site,f} - \delta^{18}O_{site,i}$	1.14‰	Tree ring $\delta^{18}O$ trends (this study)	-0.9‰	Tree ring $\delta^{18}O$ trends (this study)
$r_{E::P}$	0.4	[26]	0.325	[26]
Condensation temperature	10 ºC	[27]	10ºC	[27]
Temperature dependent fractionation factor	1.010729	[28]	1.010729	[28]

*See Supplementary Figure 3 for geographic locations and Supplementary Table 4

Supplementary Table 2. Leaf evaporative enrichment models describe fractionation occurring at the leaf level and during cellulose synthesis.

Calculation of leaf water enrichment at the sites on evaporation in the mesophyll

Assuming that $\delta^{18}O_{air} = \delta^{18}O_{sw}$ ‰, following [29],

$\mathbf{\Delta^{18}O_{es} = \varepsilon_k \left(\frac{e_i - e_a}{e_i}\right) + \varepsilon^+}$ ‰

where

$\varepsilon^+ = 2.644 - 3.206\left(\frac{10^3}{T_K}\right) + 1.534\left(\frac{10^6}{T_K{}^2}\right) \sim 9.57‰$ is the temperature dependent equilibrium fractionation[30]

$T_K = T_{leaf} = 299 \pm 4$ Kelvin; $\varepsilon_k = \frac{32 g_s^{-1} + 21 g_b^{-1}}{g_s^{-1} + g_b^{-1}} \sim 26.5 \pm 5‰$ is the leaf temperature[31], assumed to be the same as air temperature;

e_a is the ambient vapor pressure deficit, calculated from vapor pressure observations from CRU TS 4.04;

e_i is the leaf internal saturated vapor pressure, calculated from temperature[28].

$gb = 0.0105\left(\frac{U}{W}\right)^{-0.5} = 1.2 \pm 0.5\ \mathrm{mol\ m^{-2} s^{-1}}$ is the leaf boundary layer conductance to water[32,33];

$U = 0.5\ \mathrm{m\ s^{-1}}$is the wind speed (assumed);

$W = 0.04$ m (*Cedrela odorata*) and $W = 0.01$ m (*Macrolobium acaciifolium*) and leaf lamina widths.

The average δ^{18}O of leaf lamina water is then calculated as [1]

$\mathbf{\Delta^{18}O_{lw}} = \frac{\mathbf{\Delta^{18}O_{es}}(1 - e^{-\wp})}{\wp}$ ‰,

Where

$\wp \equiv \frac{Advection}{Diffusion} = \frac{u}{D/L}$;

$L = 2.36 * 10^{-5} * E^{-1.2}$ is the path length from the sites of evaporation to the leaf vein through the mesophyll intercellular spaces [34] ;

$D = 2.3 * 10^{-9}\ \mathrm{m^{-2} s^{-1}}$ is the diffusivity of $H_2{}^{18}O$ in water;

$u = \frac{E}{C}\ \mathrm{m^2 s^{-1}}$, where:

$C = 55 * 10^3\ \mathrm{mol\ m^{-3}}$

$E = g_s \frac{VPD}{atm} \sim 0.003\ \mathrm{mol\ m^{-2} s^{-1}}$ is the leaf transpiration rate;

$VPD = e_i - e_a$ is the leaf-to-air vapor pressure deificit, calculated from local climate data;

atm is the atmospheric pressure at the surface level.

g_s is the leaf stomatal conductace, which is unknown. For the purposes of this modelling we then used a measure of g_s which is sensitive to variations in VPD from its long term mean (VPD_0):

$\mathbf{g_s = g_{s0}\left(\frac{1}{1 + \frac{VPD}{VPD_0}}\right) \sim 0.2 \pm 1\ mol\ m^{-2} s^{-1}}$ (assumed)

Finally, the δ^{18}O of tree ring cellulose $\delta^{18}O_{TR}$ is then calculated as

$\mathbf{\Delta^{18}O_{TR} = (\Delta^{18}O_{lw})(1 - p_x p_{ex}) + \varepsilon_{wc}}$ ‰ [1],

where

$\varepsilon_{wc} = 27‰$ is the average fractionation factor of oxygen atoms exchanged between carbonyl groups an water during cellulose synthesis[1,5]

$p_e p_{ex} = 0.25$ is product of the the proportion of exchangeable oxygen in cellulose (p_{ex}) and the proportion of unenriched (xylem) source water in the developing cell (p_x) [1]

Supplementary References

1. Barbour, M. M. Stable oxygen isotope composition of plant tissue: A review. *Functional Plant Biology* **34**, 83–94 (2007).
2. Kahmen, A. *et al.* Cellulose δ18O is an index of leaf-to-air vapor pressure difference (VPD) in tropical plants. *Proc Natl Acad Sci U S A* **108**, 1981–1986 (2011).
3. Farquhar, G. D., Cernusak, L. A. & Barnes, B. Heavy water fractionation during transpiration. *Plant Physiology* vol. 143 11–18 Preprint at https://doi.org/10.1104/pp.106.093278 (2007).
4. Farquhar, G. & Lloyd, J. Carbon and oxygen isotope effects in the exchange of carbon dioxide between terrestrial plants and the atmosphere. in *Stable isotopes and plant carbon–water relations* (eds. Ehleringer, J., Hall, A. & Farquhar, G.) 47–70 (Academic Press, San Diego, 1993).
5. Sternberg, L. D. S. L. O. R. Oxygen stable isotope ratios of tree-ring cellulose: The next phase of understanding. *New Phytologist* **181**, 553–562 (2009).
6. Dongmann, G., Nürnberg, H. W., Förstel, H. & Wagener, K. On the Enrichment of H2180 in the Leaves of Transpiring Plants. *Rad. and Environm. Biohys.* **11**, 41–52 (1974).
7. Cintra, B. B. L. *et al.* Contrasting controls on tree ring isotope variation for Amazon floodplain and terra firme trees. *Tree Physiol* **39**, 845–860 (2019).
8. Cintra, B. B. L. *et al.* Tree-ring oxygen isotopes record a decrease in Amazon dry season rainfall over the past 40 years. *Clim Dyn* **59**, 1401–1414 (2022).
9. Brienen, R. J. W., Helle, G., Pons, T. L., Guyot, J. L. & Gloor, M. Oxygen isotopes in tree rings are a good proxy for Amazon precipitation and El Niño-Southern Oscillation variability. *Proc Natl Acad Sci U S A* **109**, 16957–16962 (2012).
10. Baker, J. C. A. *et al.* What drives interannual variation in tree ring oxygen isotopes in the Amazon? *Geophys Res Lett* **43**, 11,831-11,840 (2016).
11. Franks, P. J. *et al.* Sensitivity of plants to changing atmospheric CO2 concentration: From the geological past to the next century. *New Phytologist* vol. 197 1077–1094 Preprint at https://doi.org/10.1111/nph.12104 (2013).
12. Sprenger, M., Tetzlaff, D. & Soulsby, C. Soil water stable isotopes reveal evaporation dynamics at the soil-plant-atmosphere interface of the critical zone. *Hydrol Earth Syst Sci* **21**, 3839–3856 (2017).
13. Moreira, M. Z. *et al.* Contribution of transpiration to forest ambient vapour based on isotopic measurements. *Glob Chang Biol* **3**, 439–450 (1997).
14. Gat, J. R. & Matsui, E. Atmospheric water balance in the Amazon Basin: an isotopic evapotranspiration model. *Journal of Geophysical Research: Atmospheres* **96**, 13179–13188 (1991).
15. Cernusak, L. A., Wong, S. C. & Farquhar, G. D. Oxygen isotope composition of phloem sap in relation to leaf water in Ricinus communis. *Functional Plant Biology* **30**, 1059–1070 (2003).
16. Sternberg, L. & Ellsworth, P. F. V. Divergent biochemical fractionation, not convergent temperature, explains cellulose oxygen isotope enrichment across latitudes. *PLoS One* **6**, (2011).
17. Dansgaard, W. Stable isotopes in precipitation. *Tellus* **16**, 436–468 (1964).
18. Risi, C., Bony, S. & Vimeux, F. Influence of convective processes on the isotopic composition (δ18O and δD) of precipitation and water vapor in the tropics: 2. Physical

interpretation of the amount effect. *Journal of Geophysical Research Atmospheres* **113**, (2008).
19. Aggarwal, P. K. *et al.* Proportions of convective and stratiform precipitation revealed in water isotope ratios. *Nat Geosci* **9**, 624–629 (2016).
20. Funatsu, B. M. *et al.* Assessing precipitation extremes (1981–2018) and deep convective activity (2002–2018) in the Amazon region with CHIRPS and AMSU data. *Clim Dyn* **57**, 827–849 (2021).
21. Haghtalab, N., Moore, N., Heerspink, B. P. & Hyndman, D. W. Evaluating spatial patterns in precipitation trends across the Amazon basin driven by land cover and global scale forcings. *Theor Appl Climatol* **140**, 411–427 (2020).
22. Pattnayak, K. C., Tindall, J. C., Brienen, R. J. W., Barichivich, J. & Gloor, E. Can We Detect Changes in Amazon Forest Structure Using Measurements of the Isotopic Composition of Precipitation? *Geophys Res Lett* **46**, 14807–14816 (2019).
23. Cintra, B. B. Spatial-temporal reconstruction of Amazon flood pulse and dry season length over the past century using tree rings and isotopes of floodplain tree species Macrolobium acaciifolium. (University of Leeds, Leeds, 2019).
24. Eva, H. D. *et al.* A proposal for defining the geographical boundaries of Amazonia. https://forobs.jrc.ec.europa.eu/amazon (2005).
25. Natural Earth. Coastline. https://www.naturalearthdata.com/downloads/10m-physical-vectors/10m-coastline/ (2004).
26. Baker, J. C. A. & Spracklen, D. V. Divergent Representation of Precipitation Recycling in the Amazon and the Congo in CMIP6 Models. *Geophys Res Lett* **49**, (2022).
27. Andreae, M. O. *et al.* Aerosol characteristics and particle production in the upper troposphere over the Amazon Basin. *Atmos Chem Phys* **18**, 921–961 (2018).
28. Horita, J. & Wesolowski, D. J. Liquid-vapor fractionation of oxygen and hydrogen isotopes of water from the freezing to the critical temperature. *Geochim Cosmochim Acta* **58**, 3425–3437 (1994).
29. Dongmann, G., Nürnberg, H. W., Förstel, H. & Wagener, K. On the enrichment of H2 18O in the leaves of transpiring plants. *Radiat Environ Biophys* **11**, 41–52 (1974).
30. Bottinga, Y. & Craig, H. Oxygen isotope fractionation between CO2 and water, and the isotopic composition of marine atmospheric CO2. *Earth Planet Sci Lett* **5**, 285–295 (1969).
31. Farquhar, G. D., Ehleringer, R. & Hubic, K. T. CARBON ISOTOPE DISCRIMINATION AND PHOTOSYNTHESIS. (1989) doi:1040-2519/89/0601-503.
32. Motzer, T., Munz, N., Küppers, M., Schmitt, D. & Anhuf, D. Stomatal conductance, transpiration and sap flow of tropical montane rain forest trees in the southern Ecuadorian Andes. *Tree Physiol* **25**, 1283–1293 (2005).
33. McDermitt, D. K. Sources of error in the estimation of stomatal conductance and transpiration from porometer data. *HortScience* **25**, 1538–1548 (1990).
34. Song, X., Barbour, M. M., Farquhar, G. D., Vann, D. R. & Helliker, B. R. Transpiration rate relates to within- and across-species variations in effective path length in a leaf water model of oxygen isotope enrichment. *Plant Cell Environ* **36**, 1338–1351 (2013).
